# The Impact of Modern Anti-Diabetic Treatment on Endothelial Progenitor Cells

**DOI:** 10.3390/biomedicines11113051

**Published:** 2023-11-14

**Authors:** Velimir Altabas, Jelena Marinković Radošević, Lucija Špoljarec, Stella Uremović, Tomislav Bulum

**Affiliations:** 1 Department of Endocrinology, Diabetes and Metabolic Diseases, Sestre Milosrdnice University Clinical Hospital, 10000 Zagreb, Croatia; 2 School of Medicine, University of Zagreb, 10000 Zagreb, Croatia; 3 Srebrnjak Children’s Hospital, 10000 Zagreb, Croatia; 4 Vuk Vrhovac University Clinic for Diabetes, Endocrinology and Metabolic Diseases, Merkur University Hospital, 10000 Zagreb, Croatia

**Keywords:** endothelial progenitor cells, diabetes, diabetes complications, diabetes treatment

## Abstract

Diabetes is one of the leading chronic diseases globally with a significant impact on mortality. This condition is associated with chronic microvascular and macrovascular complications caused by vascular damage. Recently, endothelial progenitor cells (EPCs) raised interest due to their regenerative properties. EPCs are mononuclear cells that are derived from different tissues. Circulating EPCs contribute to regenerating the vessel’s intima and restoring vascular function. The ability of EPCs to repair vascular damage depends on their number and functionality. Diabetic patients have a decreased circulating EPC count and impaired EPC function. This may at least partially explain the increased risk of diabetic complications, including the increased cardiovascular risk in these patients. Recent studies have confirmed that many currently available drugs with proven cardiovascular benefits have beneficial effects on EPC count and function. Among these drugs are also medications used to treat different types of diabetes. This manuscript aims to critically review currently available evidence about the ways anti-diabetic treatment affects EPC biology and to provide a broader context considering cardiovascular complications. The therapies that will be discussed include lifestyle adjustments, metformin, sulphonylureas, gut glucosidase inhibitors, thiazolidinediones, dipeptidyl peptidase 4 inhibitors, glucagon-like peptide 1 receptor analogs, sodium-glucose transporter 2 inhibitors, and insulin.

## 1. Introduction

The endothelium is the innermost cell layer of the blood vessel wall, with distinct metabolic features that are critical in maintaining vascular integrity, regulating vascular tone, blood flow, and preventing thrombosis [1]. Under certain conditions, the endothelium may become damaged and dysfunctional, resulting in inappropriate vasomotion, inflammation, and atherosclerotic plaque build-up [2]. Atherosclerotic plaques compromise the blood supply to downstream tissues, either by vessel narrowing or by total vessel obstruction. Consequently, ischemia or infarction may occur leading to end-organ dysfunction and failure. If vital organs are affected, the most dramatic outcome is death. From the epidemiological viewpoint, atherosclerosis and its complications are the leading cause of death worldwide and represent a global burden to human health [3].

Numerous risk factors related to atherosclerosis have been identified so far; the most important include age, gender and genetics, lifestyle factors (such as smoking and diet), and underlying medical conditions, like arterial hypertension, dyslipidemia, diabetes, and obesity [4,5]. Over time, complex strategies to treat atherosclerosis and vascular events have been developed, mainly targeting already-mentioned risk factors, blood clotting, and end-organ protection [3]. Among various approaches to treat atherosclerosis and its deleterious outcomes, none of them were able to restore vascular function completely and reverse atherosclerosis. The progressive nature of this disease and the limitations of currently available treatment options lead to the general opinion that atherosclerosis is an irreversible process, strongly linked to aging [6,7].

Endothelial progenitor cells (EPCs) have recently raised significant interest since these cells can aid in regenerating the damaged vessels’ endothelium and, therefore, restore vascular function. Discovered at the end of the 20th century, they gave new insight into the pathology of atherosclerosis and offered new prospects in curative medicine [8]. EPCs are usually defined as multipotent stem cells with angiogenic potential. These cells are commonly quantified from blood by flow cytometry as mononuclear cells expressing CD34 and VEGFR2+. CD133+ and CD45−/dim are proposed as additional markers for circulating EPCs [9]. The population of EPCs is rather small, with a proportion of up to 0.05% of mononuclear blood cells [10]. Since the proportion of EPCs in blood cells is very low, some technical considerations need to be highlighted. The analysis of rare cells may be compromised with background noise. Therefore, special attention needs to be given to several preanalytical and analytical steps. These include appropriate blood sampling, collection tube choice and handling temperature, the choice of appropriate flow cytometers, erythrocyte depletion and wash/no wash protocols, background control, etc. [9]. However, to determine the proliferation capacity and the ability to form blood vessel-like forms, it is crucial to cultivate EPCs. In cultures, it became apparent that the phenotype and function of EPCs are heterogeneous, and that the cell markers differ from those in EPCs detected by flow cytometry. Originally, these subpopulations were named after the time they appeared in cultures as early and late outgrowth endothelial cells. These cells express distinct cell markers and behave diversely in cultures. They have different abilities to act as paracrine producers or to proliferate and differentiate into mature endothelial cells, contributing to the formation of new blood vessels or repairing the damaged ones. Therefore, the cells were renamed into myeloid angiogenic cells (MACs) and endothelial colony-forming cells (ECFCs). These EPC subpopulations act synergistically in vivo [11,12,13]. It is noteworthy, that there are also technical challenges in stem cell cultivation in general that may have an impact on EPC cultivation, like sample preparation, cell isolation and purification, seeding density, choice of culture media, etc. [14]. The main characteristics of these EPC subtypes isolated in culture are shown in Table 1. 

Due to their unique angiogenic features, EPCs seem to be a promising tool to treat conditions like atherosclerosis and atherosclerosis-related conditions such as ischemic heart disease, peripheral artery disease, and diabetic vascular complications [15,16,17]. In various research, improved parameters of EPC biology (e.g., increased EPC blood count quantified by flow cytometry or improved EPC function in cultures) could be correlated with improved flow-mediated dilation, brachial ankle index, or intima media thickness [18,19,20]. Furthermore, EPC biology was found to correlate with clinical outcomes like limb amputation, stroke, myocardial infarction, or death [21,22,23,24].

EPC-based therapies aim to enhance vascular repair and promote the growth of new blood vessels, ultimately improving blood flow to ischemic tissues, but there are challenges to be addressed. These include improving methods for EPC isolation and expansion, enhancing their engraftment and survival in target tissues, and optimizing the timing and delivery of EPC-based therapies [25,26].

It is noteworthy that many currently prescribed drugs have proven beneficial effects on EPC biology, like several antihypertensive drugs, statins, various classes of anti-diabetic medications, some hormones, bisphosphonates, and others [27]. Since at least some of these drugs are proven to decrease the incidence of cardiovascular incidents and mortality, it may be assumed that improved EPC biology may contribute to this effect [28]. In addition, the EPC count in the bloodstream assessed by flow cytometry or altered behavior of EPCs detected in culture can also serve as biomarkers for cardiovascular health. Reduced EPC numbers or impaired EPC function are associated with various cardiovascular diseases, correlating with advanced disease stage and response to treatment [22]. Understanding the biology of EPCs, and the way they react to currently available pharmacologic interventions is essential for unlocking their full potential in clinical practice [29].

The aim of this review is to discuss the impact of modern anti-diabetic treatment on EPC-mediated vascular repair, correlate it to proven clinical effects on vascular health, and discuss the potential effect of EPCs on recent cardiovascular outcome trial results.

## 2. Endothelial Progenitor Cells in Health and Disease

There are established regenerative cell responses identified that can diminish and even heal vascular injury and re-establish endothelial integrity and function. A prominent role in vascular repair in adult humans is had by resident endothelial progenitor cells, located in the blood vessel wall. These cells may replicate and differentiate into mature endothelial cells in response to vascular injury. In contrast to resident endothelial progenitor cells present in blood vessels’ walls, another type of endothelial progenitor cells may be found in distant tissues like bone marrow, fat tissue, and the spleen. EPCs from distant locations may be mobilized into the bloodstream under certain conditions like ischemia and hypoxia. Under conditions with limited oxygen supply, hypoxia-induced factor 1 (HIF-1) is released, thus inducing stromal-derived factor 1 (SDF-1) production and release. SDF-1 is the key regulator of EPC mobilization. SDF-1 interacts with other mobilizing factors like VEGF and E-selectin, and the PI3K/Akt/eNOS-dependent signal transduction pathway, leading to mobilization of progenitor cells into the blood flow. In addition, there are certain enzymes involved in this EPC mobilization like matrix metalloproteinase 8 and 9 (MMP-9) that inactivate retention factors at the site of EPCs’ origin [30,31]. Blood glucose, erythropoietin, thyroid hormones, and estrogen may modulate EPC mobilization [11,27,32]. 

EPCs enter the blood circulation, migrate to the place of vessel injury, embed there in a process named homing, and produce various paracrine substances like SDF-1, nitric oxide (NO), vascular endothelial growth factor (VEGF), fibroblast growth factor (FGF), insulin-like growth factor (IGF), and others. SDF-1 leads to upregulation of its specific chemokine receptor type 4 (CXCR-4 receptor) on the EPC surface, resulting in enhanced homing of these cells to the injured blood vessel. Other adhesion molecules, like E-selectin, integrins, intercellular adhesion molecule (ICAM), and vascular cell adhesion molecule (VCAM) are also included in this process [30]. Responding to paracrine factors secreted by embedded circulatory EPCs like VEGF, IGF-1, and gasotransmitters, resident endothelial progenitor cells proliferate and differentiate in situ resulting in vascular repair [11,30,32]. The AMPK/Akt/eNOS and AMPK/eNOS signaling pathways with their activators (adipokines, prostaglandins) have an important role in enhancing further EPC differentiation and vessel formation [33,34]. 

As mentioned earlier, circulating EPCs represent a heterogeneous cell population with varying characteristics. Two types of EPCs could be detected in the circulation and characterized after in vitro cultivation: MACs or early-onset EPCs, and ECFCs, known also as late-onset EPCs. MACs form colonies in cultures under special conditions only, and ECFCs are better differentiated and provide mature endothelial cells as building blocks important for physical endothelial integrity and function. Both progenitor cell types can produce paracrine substances like nitric oxide (NO) and express receptors for growth factors like the vascular endothelial growth factor receptor 2 (VEGF-R2) [35,36,37]. 

In healthy individuals, both resident and circulating EPCs contribute to the maintenance of vascular health by continuously replenishing and repairing the endothelial lining [38]. Considering the impact of circulating EPCs on vascular repair, it seems that their paracrine function is more important since the resident progenitor cells provide the majority of newly differentiated cells necessary for vascular repair [11,39].

It has to be noticed that the turnover of healthy human endothelial cells is rather low in regions with laminar blood flow, with a cell lifespan of many years [40]. However, in regions with turbulent blood flow, like vessel curvatures and branching points, the lifespan of endothelial cells may be shortened and cell turnover increased. In humans, the endothelial turnover is within the range from 47 to 23,000 days [41,42]. Endothelial damage may occur over time due to various mechanical and chemical stimuli. Mechanical factors like increased blood pressure may directly harm the endothelium, while chemical factors like hyperglycemia or smoking may trigger premature endothelial cell apoptosis. All these mentioned factors disrupt endothelial integrity, resulting in an elevated mature endothelial cell count in peripheral blood, as a marker of endothelial damage. Without the functional innermost layer, NO production is compromised, and vascular tone and blood flow become dysregulated, resulting in endothelial dysfunction and downstream tissue ischemia. In addition, a cascade of inflammatory processes on the site of injury triggers cholesterol accumulation and oxidation promoting further growth of atherosclerotic plaques. Furthermore, cytokines secreted by the damaged tissue attract smooth muscle cells that migrate from the artery’s muscle layer into the vascular intima. These cells further proliferate leading to plaque build-up. Oxidized cholesterol attracts macrophages, further contributing to a pro-inflammatory environment. The plaque surface is covered by a fibrous cap that may become unstable in a pro-inflammatory environment and finally rupture, leading to activation of the clotting cascade, resulting in vessel obstruction and infarction of related tissues [43,44]. 

In conditions where blood flow is compromised, such as in ischemic diseases (e.g., peripheral artery disease or myocardial infarction), repairing mechanisms are activated: EPCs from distant tissues enter the circulation, providing paracrine factors from myeloid angiogenic cells to stimulate both embedded late outgrowth endothelial progenitor cells and resident endothelial progenitor cells, leading to vascular repair [11,36]. In that way, the presence of EPCs helps restore blood flow by contributing to the formation of collateral vessels, which bypass blocked or narrowed arteries.

Functional EPCs decrease the risks of thrombotic events. This occurs either by replacing aged and injured endothelial cells with EPCs or by releasing substances that inhibit blood clot formation from EPCs to an extent that is still under debate [45,46]. The number and function of EPCs decline with age, which can contribute to the development of age-related vascular diseases, such as atherosclerosis. Gender-specific differences with a protective higher estrogen-dependent EPC count in fertile women during the ovulatory phase compared to age-matched men have been found [47]. EPC dysfunction is also implicated in various other related pathologies, including diabetes, dyslipidemia, and arterial hypertension, highlighting their significance in vascular disease progression [46,48].

## 3. Diabetes and Endothelial Progenitor Cells Biology

Diabetes mellitus is a metabolic disorder characterized by chronically elevated blood glucose levels. Depending on the diabetes type, deficient insulin action and insulin resistance may additionally contribute to the occurrence of other metabolic abnormalities like dyslipidemia and abnormal protein metabolism, resulting in inflammation and oxidative stress [49]. These metabolic alterations result in endothelial dysfunction, a key initiating event in vascular complications. Furthermore, diabetes is often accompanied by other conditions affecting endothelial function and vascular health like obesity, arterial hypertension, and dyslipidemia [50]. In addition, diabetes is associated with changes in the coagulation cascade, platelet function, and fibrinolysis, creating a prothrombotic state and increasing the risks of thrombotic events [51]. Over time, chronic diabetic complications may develop. These conditions are linked to vascular dysfunction and structural damage, affecting both large and small blood vessels in the body [52].

An important pathophysiological mechanism linking diabetes and its complications seems to be related to both MACs and ECFCs. Namely, hyperglycemia, hypoglycemia, and increased glucose variability occurring in diabetic patients, regardless of diabetes type, have detrimental effects on EPC biology [53]. Patients with both type 1 and type 2 diabetes have reduced numbers of circulating EPCs. This decrease can be noticed early after the diagnosis of type 1 and type 2 diabetes, regardless of patients’ age [54,55]. Additional studies have shown that in individuals with diabetes, EPCs often exhibit reduced mobilization capacity, shortened survival, and impaired ability to differentiate into mature endothelial cells [37,53,56,57,58]. 

**Figure 1:**
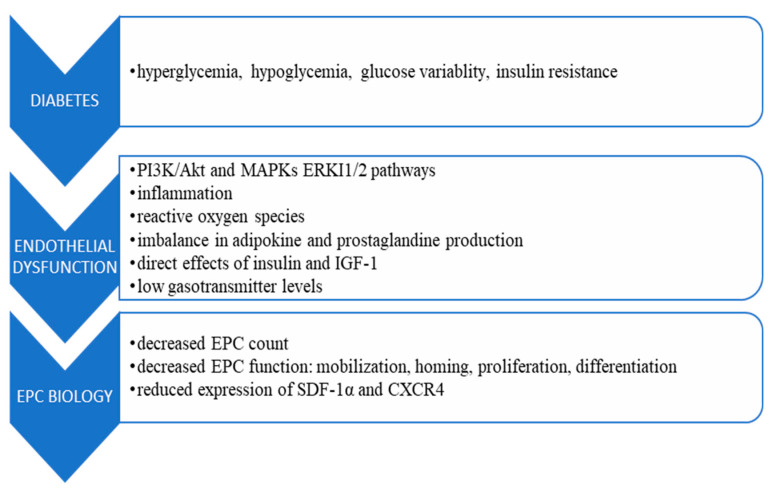
Mechanisms of endothelial dysfunction in diabetes [27,53,54,55,57,59,60,61,64,65,72,73,74,75,76,77,78,79,80,81,82,83,84].

Aside from the specific metabolic environment determined primarily by blood glucose abnormalities, possible mechanisms involved in the deterioration of EPCs abilities for vascular repair include disruptions in NO pathways, impaired intracellular signaling in other pathways (MAPK/ERK, SDF-1/CXCR-4) and the p53/sirtuin1 (SIRT1)/p66Shc axis, inflammation, reactive oxygen species, accumulation of advanced glycation end products (AGE), low levels of gasotransmitters, imbalance in adipokine production, and direct effects of insulin and IGF-1, as shown in Figure 1 [27,53,59,60,61]. In particular, MAPK causes NF-κB-dependent inflammatory stress response in the bone marrow, disrupting hematopoietic progenitor activity and enhancing inflammation-induced hypoxic injury [62], while p53/sirtuin/p66Shc disruption promotes EPC senescence [63]. Reactive oxidative species impair EPCs viability, increase apoptosis, and negatively impact tube formation, and AGE accumulation disrupts NO production and significantly decreases anti-oxidant enzymes, thus increasing oxidative stress [64]. Impaired gasotransmitter function, including decreased NO, CO, and H_2_S signaling, may alter extracellular matrix properties, affecting metalloproteinase function [65]. 

EPC dysfunction results in compromised function of both large and small blood vessels and contributes to the development and progression of diabetes-related vascular complications [53,66,67]. It has been shown that EPC dysfunction is closely linked to the occurrence of diabetic microvascular complications, such as retinopathy, nephropathy, and neuropathy [66,68,69]. These complications may lead over time to vision impairment and blindness, kidney function decline and end-stage renal disease, altered or loss of sensation in the extremities, and delayed wound healing, and are therefore leading causes of invalidity in developed countries [70,71]. In other words, enhanced EPC-mediated angiogenesis and endothelial repair in the renal, retinal, and peripheral vasculature could contribute to better outcomes in diabetic individuals. 

EPC dysfunction is also implicated in macrovascular diabetic complications, including coronary artery disease, cerebrovascular disease, and peripheral artery disease. Diminished EPC count and function contribute to impaired endothelial repair mechanisms, leading to endothelial dysfunction and accelerated plaque formation, which increase the risk of major cardiovascular events (MACEs) in diabetic individuals. Even more serious, a decreased EPC function is linked to premature mortality [18,72]. It has been proposed that EPC levels and function could serve as valuable biomarkers for diabetes-related complications. Monitoring EPC parameters may help identify individuals at higher risk of developing vascular complications, allowing early intervention and personalized treatment strategies [22]. However, it is important to note that individual patient factors, including age and the presence of comorbidities, can influence the response of EPCs to treatment and modify their effect on the endothelium [6,46]. Therefore, personalized medicine approaches that consider these factors may be necessary to optimize diabetes management.

Several landmark studies have shown that good blood glucose control decreases the risk of developing chronic diabetic complications and mortality [73,74]. Achieving good blood glucose control requires, in general, both non-pharmacological interventions and drug therapy. Recently, some anti-diabetic medications like sodium–glucose transporter-2 inhibitors and glucagon-like peptide-1 (GLP-1) receptor agonists have proven to have better cardiovascular outcomes in patients with type 2 diabetes, and these medications were highlighted in modern international guidelines [75]. 

## 4. Currently Available Diabetes Treatments Affecting EPC Count and Function

### 4.1. Lifestyle Modification

Lifestyle modifications are integral components of diabetes management. Most importantly, evidence-based strategies encompass dietary adjustments, appropriate exercise programs, body weight optimization, smoking cessation, and prudent alcohol choices. A healthy lifestyle has been proven to benefit blood glucose control and, thus, help to reduce diabetic complications in patients with diabetes [76,77]. Nutrition therapy is an umbrella term used for dietary modifications in diabetes therapy. Quality nutrition choices have been shown to improve blood glucose control and, thus, help to reduce diabetic complications [78,79,80,81,82]. At the time, to our best knowledge, there is a single published paper on a trial regarding EPCs in diabetic patients with a focus on nutrition. The authors found a beneficial effect of a Mediterranean diet compared to a low-fat diet on EPC count (CD34+VEGFR2+ and CD34+VEGFR2+CD133+) and carotid intima-media thickness in patients with type 2 diabetes. The increased EPC count correlated with improvements in blood glucose control, insulin sensitivity, total cholesterol, high-density lipoprotein cholesterol, and systolic blood pressure, decreasing the risk for chronic diabetic complications [83].

Several other studies focused on non-diabetic study populations with risk factors for cardiovascular events or on healthy volunteers. However, because of the complex interplay of diabetes, other risk factors, and cardiovascular disease, these trials are also relevant for diabetic macrovascular complications. Adding vegetables to the diet improved EPC counts in a randomized small study in Japan involving healthy volunteers. From a contemporary viewpoint, a serious limitation of the study is the lack of a precise EPC definition [84]. Better EPC (CD34+/VEGFR2+) counts and improved vasomotor function measured by flow-mediated vasodilation on the brachial artery were detected when flavanols were added to the diet of patients with coronary artery disease [85]. The Mediterranean diet, rich in vegetables and unsaturated fats also increased EPC (CD34+/VEGFR2+/CD133+) counts and reduced endothelial cell microparticles related to mature EC apoptosis in a small group of elderly people with a clinical correlate of improved ischemic reactive hyperemia, indicating reduced endothelial damage and improved endothelial regenerative capacity [86]. Similar results, like improved EPC (CD34+/VEGFR+/CD133+) counts, a decreased level of circulating microparticles, an improved anti-inflammatory profile, and increased flow-mediated vasodilation were found in the larger CORDIOPREV trial involving patients with already diagnosed coronary artery disease who were put on the Mediterranean diet. A simplified dietary approach with fat restriction was statistically inferior in this trial [87]. Calorie restriction in combination with the Mediterranean diet and exercise also had beneficial effects on EPC (CD34+/VEGFR2+) count in patients with metabolic syndrome after three months of intervention. Insulin sensitivity and blood pressure were also improved and body weight loss was promoted. However, endothelial function was improved only in subjects randomized to exercise [88]. Adding polyunsaturated fat from fish was also shown to improve both circulatory EPC (CD34+/VEGFR+; CD133+) count and tube-formation function after a 6-week intervention, but this effect ceased in the following 6 weeks after the intervention was stopped. An improvement in several inflammatory substances like TNF α, interleukin 8 (IL-8), and an improvement in adhesion molecule profile was also detected [89]. In another randomized trial, replacing saturated with monounsaturated and polyunsaturated dietary fats in a larger sample of people at moderate risk for cardiovascular disease increased EPC (CD34+/VEGFR2+) and decreased microparticle numbers, suggesting beneficial effects on endothelial repair and maintenance [90]. Intriguingly, excluding meat and fish from nutrition showed less favorable effects on EPC (CD34+/CD133+/CD45−/dim) than adhering to the full Mediterranean diet in the CARDIVEG study [91]. An ancient type of grain also showed an increase in EPCs (defined as CD34+ or CD133+ cells) in comparison to modern grain varieties after an intervention of 8 weeks. At the same time, significantly better blood glucose control and cholesterol levels were achieved [92].

Besides the approach with the Mediterranean diet, green tea may improve the EPC count (CD34+/VEGFR2+/CD45−/dim) even over the short term in a population of chronic smokers. In the same period, an improvement of vascular function assessed by flow-mediated dilation was detected. However, since there was no control group in the study, definitive conclusions cannot be made [93]. Wise alcohol choices may also benefit EPC biology. In a trial with a cross-over design, the effect of beer and non-alcoholic beer on circulating EPC (CD34+/VEGFR2+/CD133+) levels was tested in patients with high cardiovascular risk, compared to gin. The beer and non-alcoholic beer increased the circulating EPC count and SDF-1 in the peripheral blood. On the other hand, while the same subjects were drinking gin during the control period, there was a decrease in EPC count. A major study limitation is the lack of a control group drinking water [94]. Considering other types of alcohol, red wine has been shown to increase circulating EPC (CD34+/VEGFR2+/CD133+) levels and to improve EPC function (attenuated senescence and improved adhesion, migration, and tube formation) by modifying nitric oxide bioavailability, at least in healthy volunteers who were randomized either on red wine, vodka, beer, or water. Clinically, improved FMD vasodilation was seen in patients drinking red wine [95]. White wine also increases the EPC (CD34+/VEGFR2+, CD133+) count and decreases concentrations of several pro-inflammatory markers in a population with increased cardiovascular risk, but there was also no control group on water [96]. It is unclear if alcohol is the key effect mediator since grape seeds have been shown to also improve CD34+ cell counts. A more precise EPC characterization would be necessary to confirm this result [97]. 

Essentially, dietary adjustments recommended for patients with diabetes do not only improve blood glucose control but reduce the need for pharmacotherapy. It seems that they may improve vascular health by benefitting several aspects of EPC biology like EPC count, modifying NO production, and reducing the number of endothelial microparticles in circulation. However, the best dietary approach for diabetic patients needs still to be established since there is only one study performed in this population. This study showed the benefits of the Mediterranean diet. Other approaches like adding unsaturated fat, vegetarian diet, caloric restriction, and others still need to be evaluated in diabetic patients. Correlating EPC findings with clinical tests like FMD would enhance the predictive value of such research. However, eating more vegetables, adding more unsaturated fat to the diet, and caloric restriction could positively impact EPCs in non-diabetic persons, but in some parts of these studies, there was no correlate with other clinical findings. The exact mechanisms by which food influences EPC biology remain to be elucidated, but improved insulin sensitivity, decreased inflammatory parameters, and prostaglandins could play a significant role [98,99,100,101]. Intriguingly, alcohol could not show benefits on EPC parameters, with the exception of red wine, and there could not be a definitive conclusion drawn on green tea. In addition, EPCs are not equally defined in these studies, making comparations between studies difficult. 

Exercise is another lifestyle factor that affects blood glucose control, but also cardiovascular morbidity and mortality in patients with diabetes. There are several trials dealing with the effect of exercise on EPC biology in diabetic patients. Acute exercise loads promptly increased the EPC (CD34+/CD133+) count in patients with type 2 diabetes with and without nephropathy [102]. In patients with type 2 diabetes and diagnosed coronary artery disease, an elevated level of microparticles and EPC could be detected. In contrast, patients with type 1 diabetes have shown a blunted EPC response to both aerobic and resistance exercise. The authors concluded that these findings may suggest a low reserve of EPC (CD34+/CD45dim and CD34+/VEGFR2+/CD45dim) in the bone marrow in patients with type 1 diabetes [103]. These findings have been confirmed by another trial by an independent group of authors, testing CD34+/VEGFR+ and CD34+/VEGFR2*/CD45dim EPC count [104]. More studies have shown the impact of exercise in non-diabetic populations, with or without other cardiovascular risk factors. Studied populations include healthy volunteers, obese adolescents, patients with renal failure, patients with heart failure, and patients with coronary artery disease [105,106,107,108,109]. In general, moderate exercise increases the EPC count, improves their migratory capacity, and reduces EPC apoptosis, with beneficial effects on vascular healing. Exercise may influence EPC counts and EPC function by several mechanisms that include increased NO production, improved insulin sensitivity, decreased inflammation, and improved hormonal and cytokine signaling [110,111]. However, EPCs are not universally equally defined in these research, making direct comparison hard.

Finally, an important lifestyle factor affecting diabetic and vascular outcomes is smoking. While nicotine is responsible for tobacco addiction, cigarette smoke contains many substances resulting from tobacco combustion. Oxidizing chemicals, carbon monoxide, particulates, heavy metals, nitrosamines, and polycyclic aromatic hydrocarbon carcinogens are drivers of tobacco toxicity and may affect EPC biology. One important mechanism responsible for endothelial damage is compromised tetrahydrobiopterin depletion leading to decreased NO availability, endothelial dysfunction, increased inflammation, and activation of platelets, resulting in accelerated atherogenesis [112]. Smokers are shown to have decreased EPC (CD34+/VEGFR2+) counts and impaired endothelial-dependent vasodilatation compared to non-smokers. The survival of EPC is also shortened [113]. Both active and passive smoking impact EPC biology negatively. Smoking cessation increases EPC (CD34+/VEGFR2+/CD133+) count and function by reducing hypoxia, reactive oxygen species (ROS) generation, and inflammation [113]. Intriguingly, consuming electronic cigarettes increases acutely the count of CD34+/CD309+ cells in regular smokers, maybe indicating an acute bone marrow response to vascular injury. Still, there is not enough evidence to draw definitive conclusions about electronic cigarettes [114]. Important to note, these studies are not consistent in EPC characterization, and there are no studies involving diabetic patients performed up to the present time, making conclusions on this topic elusive.

### 4.2. Anti-Diabetic Agents 

Many different anti-diabetic agents are currently used to treat diabetes. Better glucose control itself is associated with improved endothelial function and a decreased risk of microvascular and macrovascular diabetic complications. There is mounting evidence about the impact of currently available anti-diabetic drugs on cardiovascular health. Furthermore, since 2008, every new anti-diabetic drug needs to be tested in a cardiovascular outcome trial if seeking approval from the Food and Drug Agency (FDA). In these trials, new agents are compared to standard treatment, and must at least prove non-inferiority considering cardiovascular outcomes. Cardiovascular outcomes of interest typically include cardiovascular death, non-fatal myocardial infarction, and stroke. Optional other outcomes may be additionally considered, like total mortality, hospitalizations for heart failure, lower limb amputations, albuminuria, etc. A brief overview on anti-diabetic medications, their mode of action, cardiovascular safety/superiority, and clinical trials involving EPC biology is shown in Table 2.

Possible mechanisms of cardiovascular benefits include beneficial effects on EPC health. EPCs may benefit from improved glycemia and insulin sensitivity, enhanced nitric oxide production, reduced oxidative stress, and decreased inflammatory parameters. Some drugs improve EPC homing by other mechanisms, including altered expression of adhesion molecules. Specific effects on EPC may vary according to differences among various classes of anti-diabetic drugs. 

Among the drugs with favorable cardiovascular effects is metformin. For many years considered the first-line anti-diabetic therapy for patients with type 2 diabetes, metformin has shown cardiovascular benefits in the landmark UKPDS study [146]. Considering EPC biology, one study found that circulating EPC counts increased after metformin initiation in patients with type 2 diabetes, correlating with increased FMD [115]. Later, an increase in EPCs was proven also in patients with type 1 diabetes, when metformin was given as a supportive treatment in the MERIT trial [116]. Regarding the EPC increase with metformin in patients with type 2 diabetes, this effect could be enhanced when gliclazide, a sulphonylurea, was added [117]. It seems that the most important mechanism affecting EPC biology in metformin-treated patients is increased phosphorylated-eNOS expression and NO production in cultures, as well as altered AMPK function [147]. 

In general, the effect on EPCs of most other anti-diabetic drugs was tested when these drugs were added to metformin, with few exceptions. 

Gliclazide alone was also able to improve EPC biology, correlating with flow-mediated vessel dilatation and improvements in markers of oxidative stress as the key mechanism of action on EPCs [118]. It seems so far that gliclazide is the only sulphonylurea with a proven effect on EPCs. Glibenclamide did not show any increase in EPCs in a trial when it was compared to vildagliptin [119]. The same was shown for glimepiride when compared to sitagliptin [120]. Although hampered by methodological limitations in EPC characterization and the absence of clinical assessment of endothelial function, this finding fits perfectly into the context of better cardiovascular outcomes in patients treated with gliclazide compared to other sulphonylureas after myocardial infarction [148,149].

Another class of oral anti-diabetic drugs are thiazolidinediones, or peroxisome proliferator-activated receptors gamma (PPAR γ) agonists. Although they are used to improve blood glucose control, they have also been shown to affect other cardiovascular risk factors, like dyslipidemia and albuminuria [150,151]. Since these drugs may cause water retention, heart failure may occur [152,153]. Pioglitazone was shown to reduce the incidence of some major adverse cardiovascular events in patients with type 2 diabetes [154,155]. Two members of this class, rosiglitazone and pioglitazone, have been proven to impact EPCs. In patients with type 2 diabetes, rosiglitazone improved EPC re-endothelialization impaired by NADPH oxidase activity, diminishing the effects of oxidative stress [156]. Human studies with pioglitazone have demonstrated increased EPC counts and proliferative ability, decreased EPC apoptosis, and improvements in some metabolic parameters and inflammatory markers [121,122,123,124]. Improved adipokine profile and anti-inflammatory properties were also proposed mechanisms to obtain EPC biology improvement [122]. In some studies, CD34+/VEGFR2+ EPCs were investigated, and these studies showed favorable results on EPC health, but the clinical response on endothelial function remains unknown since it was not tested. It has to be mentioned that in one study, pioglitazone showed no impact on EPC counts, but in this study there is no evidence about the method used for EPC determination [125]. These results justify the need for future research regarding the impact of thiazolidindiones on EPCs and clinically assessed endothelial function. 

Incretin-based therapies emerged recently as interesting add-ons to diabetic pharmacotherapy. There are two classes of incretin-modulating drugs. The first one contains inhibitors of dipeptidyl peptidase-4 (DPP-4 inhibitors). The main mode of action is inhibiting the breakdown of endogenous incretins, mainly GLP-1, thus maintaining the concentration of this substance within a physiological range. This incretin then enhances food-triggered insulin secretion. The majority of them like sitagliptin, linagliptin, saxagliptin, and alogliptin were tested in cardiovascular outcome trials and were proved to be non-inferior to standard treatment [157,158,159,160]. In other words, these drugs were as good as standard treatment at that time, like metformin, gliclazide, thiazolidindiones, or insulin. The impacts of vildagliptin and teneligliptin on cardiovascular outcomes were not tested in large, randomized trials.

Considering the effect of DPP-4 inhibitors on EPCs, there are some results published showing the favorable impact of these drugs. Improved glucoregulation, improved oxidative parameters, increased NO and SDF-1 production, and decreased inflammation are the proposed mechanisms for these effects [127,128,131]. Sitagliptin increased EPCs and SDF-1 during 4 weeks as add-on treatment compared to standard treatment involving metformin and/or insulin secretagogues in a small sample of people with type 2 diabetes [126]. Similar results considering EPC count, but with a decrease in SDF-1α during a 12-week trial in patients with type 2 diabetes, were detected when sitagliptin was added to metformin [120]. These results on SDF-1 fit less well into the proposed mechanism of action of DPP-4 inhibitors since DPP-4 degrades SDF-1 [161]. More recently, sitagliptin was tested against metformin in a short trial for three days. The drug increased EPCs, but also SDF-1α and NO concentration, with the most profound impact on patients receiving both drugs [127]. In another trial, in which voglibose was used as the comparator drug, sitagliptin was shown to increase EPCs and improve flow-mediated vasodilation, while voglibose showed no effect [128]. Linagliptin also increased the EPC count acutely in patients with type 2 diabetes during a 4-day trial, with increased concentrations of SDF-1α. Intriguingly, DPP-4 activity was abated by more than 50%, indicating other mechanisms responsible for an increase in SDF-1α [129]. An increase in SDF-1 with linagliptin has been observed even after 6 months of treatment. The effect on EPCs was not statistically significant [130]. However, in patients with chronic kidney disease, linagliptin improved EPC count, the antioxidant level was enhanced, and clinical parameters like augmentation index and pulse wave parameter were improved during a 12-week trial [131].

As mentioned before, vildagliptin also increases the number of EPCs, but with a reduction in SDF-1α levels after 12 months, in comparison to glibenclamide [119]. Alogliptin showed the same effect as gliclazide on the EPC count. The authors concluded that the observed increase in EPCs seemed to be due to the glucose-lowering effect of both drugs [132]. Furthermore, saxagliptin failed to be superior to metformin in two 12-week trials. Although there was an increase in EPC count and improvement in flow-mediated dilation, metformin showed similar effects when these drugs were given as monotherapy, with no add-on effect on EPCs in dual treatment [133,134]. Finally, teneligliptin showed an increasing trend in the number of EPCs, albeit this did not reach statistical significance. However, flow-mediated vasodilatation improved in the study group, suggesting a mechanism different than EPC [135]. In conclusion, there is some evidence that DDP-4 inhibitors improve certain aspects of EPC health; the main limitation is the absence of clinical endothelial assessment in the majority of research, and a short study duration in some of the trials.

In contrast to DPP-4 inhibitors, GLP-1 receptor agonists override the physiological effects of endogenous GLP-1 and exert a stronger effect on GLP-1 receptors. These are potent anti-diabetic drugs with the main mechanism of action that stimulates insulin secretion after an oral glucose load via the incretin effect. Other medical benefits may include delaying gastric emptying, inhibiting glucagon production, decreasing pancreatic beta-cell apoptosis, promoting weight loss, lowering arterial blood pressure and total cholesterol, improving left ventricular ejection fraction, myocardial contractility, coronary blood flow, cardiac output, and endothelial function while reducing infarction size. Some of them like liraglutide, semaglutide, and dulaglutide are proven to be superior to standard anti-diabetic treatment in secondary cardiovascular prevention [162,163,164]. Interestingly, there are limited data about their effects on human EPCs. In a small trial, liraglutide was inferior to exenatide considering EPC counts, but liraglutide was not given in the full dose of 1.8 mg daily. The authors speculated about antioxidative/anti-inflammatory effects as mediators on EPC biology [136]. In a head-to-head trial, comparing liraglutide in the full dose of 1.8 mg daily and sitagliptin, there was a similar effect on EPC count, with a more favorable effect of liraglutide on VEGF and SDF-1α after 26 weeks. However, in this trial, CD 34+/VEGFR2+ EPCs were not investigated [137]. So far, dulaglutide is the single GLP-1 receptor agonist that showed an increase in EPC count and function resulting in improved clinical parameters like brachial-ankle pulse wave velocity. Lower grades of inflammation and increased NO production were the supposed mechanisms for these effects [138]. No data on humans for semaglutide have yet been published. Putting all these data into the context of previously published cardiovascular outcome trials which stated superiority for the majority of these drugs [162,163,164], and borderline significance (*p* = 0.06) for exenatide [165], it seems that further investigations are justified to prove the beneficial impact of GLP-1 receptor agonists on EPCs.

Sodium-glucose transporter-2 inhibitors (SGLT2i) are the latest introduced class of anti-diabetic treatment. Their main mode of action involves the inhibition of the sodium/glucose co-transporter-2 (SGLT-2) in the kidneys’ proximal tubule, causing glycosuria and lowering blood glucose levels independent of insulin action. Empagliflozin was the first anti-diabetic drug to show cardiovascular superiority over standard anti-diabetic treatment [166]. Later it was recognized that the benefit of the entire drug class goes beyond the glucose-lowering effects, from reducing the risk of MACEs, hospitalization for heart failure, and worsening of chronic kidney disease (CKD) in diabetic patients, to reducing the rate of cardiovascular death and hospitalization for heart failure in nondiabetic patients [167]. There is a limited number of clinical research studies considering the impact of SGLT2i on EPC biology. In the first randomized controlled trial of dapagliflozin vs. placebo with an open-label extension and an open-label observational study of empagliflozin treatment on levels of circulating stem cells (CSCs) and EPCs, results showed a non-significant increase in CSC and EPC after short-term treatment with SGLT-2is. After 1.5 years of dapagliflozin treatment, the EPC count significantly increased. The authors concluded that cardiovascular protection cannot be directly correlated with EPC counts, suggesting protection is dominantly attributable to other factors [139]. In another trial, empagliflozin increased the subpopulations of circulating cells expressing CD133+ following 6 months of treatment, while improving inflammation parameters [141]. In addition, dapagliflozin was shown to improve the vasculogenic capacity of EPCs via activating AMPK-mediated inhibition of inflammation and oxidative stress in a study comparing patients with type 2 diabetes with healthy controls over 3 months [140]. Similarly, a significant better expression of the CXCR4 receptor with an increase in the migratory function of CD34+ cells and an increase in the expression of antioxidants (superoxide dismutase 2, catalase, and glutathione peroxidase) in canagliflozin-treated patients as compared to the placebo group was shown [142]. These results suggest that the action of SGLT-2i may also be in part mediated through the effect on EPCs with consequently beneficial effects that go beyond the glucose-lowering effect, but it is still too early to deduce on the effect of this drug class on EPCs. Although there are only a few studies exploring these issues, they showed new directions in explaining and understanding the beneficial effects of SGLT-2i. Future research would surely benefit from better EPC definition.

The effect of insulin analogs on EPCs has also been investigated. In vitro, insulin can mobilize EPCs. In patients suffering from type 2 diabetes, both insulin glargine and detemir raised EPC counts, with no difference between the two drugs over 6 months [143]. However, it seems that long-acting insulin analogs increased the EPC count to a greater extent in comparison to intermediate-acting human insulin and oral drugs with a trend towards improved intima-media thickness [144]. Intensive insulin therapy enhanced EPC counts over 6 months compared to basal–oral therapy (metformin and/or sulphonylureas) in patients with type 2 diabetes and peripheral artery disease undergoing peripheral angiography and subsequent angioplasty procedure. However, the cumulative incidence of restenosis/amputation/limb salvage procedures/death in patients with type 2 diabetes and chronic limb ischemia patients did not differ between groups at the study end, but there was a significant effect on eNOS gene variants [145]. In addition, hypoglycemia during insulin therapy may negatively impact EPC biology and clinical outcomes [56]. Since there was no inherent effect of both NPH insulin and insulin glargine on EPC count compared to escalated oral therapy, it may be concluded that there is no specific effect of insulin beyond better glucose control. These findings fit into the general accepted fact that novel insulins are not superior to standard treatment in terms of reducing MACEs. Other factors, like eNOS genetic polymorphism and eventual hypoglycemic events, may easily overshadow the beneficial effects of insulin therapy.

Finally, it has to be mentioned that trials involving patients with type 1 diabetes are few. The majority of treatment discussed previously is registered for patients with type 2 diabetes. In an interesting trial involving patients with type 1 diabetes, reducing glucose variability with new technologies like insulin pumps showed beneficial effects on EPC count in a 6-month trial [58]. As mentioned before, adding metformin to insulin treatment in type 1 diabetes may improve EPC count and function [116].

## 5. Conclusions

EPCs are a captivating and vital component of the vascular system. Their roles in vascular repair, regeneration, and potential therapeutic applications make them a subject of ongoing research and hold promise for improving cardiovascular health and advancing regenerative medicine. Vessel regeneration and repairment are both altered in diabetes mellitus. Consequently, micro and macroangiopathic complications may develop. Therefore, EPCs have become the target of interest for many scientists who are putting an effort into discovering treatment options that can affect their count and function. Many available anti-diabetic drugs like metformin, sulphonylureas, PPAR γ agonists, DPP-4 inhibitors, and insulin are proven to improve, under certain conditions, the low number and functional impairment of EPCs. SGLT-2i and GLP-1 receptor agonists are the newest anti-diabetic drugs with limited evidence of beneficial effects on EPC biology. Future research will likely focus on untangling the complexity of EPC biology and developing innovative approaches to harness their full potential both in type 2 and type 1 diabetes.

To conclude, current data suggest that the low number and dysfunction of EPCs can be improved by treatment of diabetes with currently available drugs, either through drugs’ specific mechanisms or through improving blood glucose control.

## Figures and Tables

**Table 1:** Characteristics of distinct EPC subtypes in cultures [13].

Lineage	Myeloid Angiogenic Cells	Endothelial Colony-Forming Cells
positive cell markers	CD45, CD31, CD14	CD31, CD105, CD 146 VE-cadherin, von Willebrand factor, VEGFR2 CD34+/−
negative cell markers	CD146, CD34	CD45, CD14
in vitro effects	conditioned media necessary for the endothelial formation	intrinsic tube forming capacity
function	provide paracrine angiogenic factors	provide cells as building blocks release of paracrine factors
time of appearance in culture	early	late

**Table 2:** Currently available anti-diabetic medications, their main mode of action, cardiovascular effects, and effects on EPCs.

Drug Class	Mode of Action	Cardiovascular Effects	Effects on EPCs	Reference
Biguanides: Metformin	reduces hepatic glucose production facilitates peripheral glucose uptake and utilization, in part by increasing insulin action reduces basal hyperinsulinemia alters glucose turnover in the gut increases glucose uptake from circulation and decreases absorption from food increases the release of glucagon-like peptide-1 (GLP-1) alters the gut microbiome activates adenosine monophosphate-protein-kinase (AMPK) activator and increases the transport capacity of all types of membrane glucose transporters (GLUTs)	beneficial effects not proven in CVOTs	↑EPC (CD34+/VEGFR2+/CD45-/dim) count assessed by flow cytometry ↑FMD	[115]
			↑EPC (CD34+/VEGFR2+/CD45-/dim) count assessed by flow cytometry ↑ECFC colonies number ↑adhesion capability of proangiogenic cells using fibronectin adhesion assay	[116] *
			↑EPC (CD34+/VEGFR2+/CD45-/dim) count assessed by flow cytometry	[117]
1Sulphonylureas: Gliclazide Glimepiride Gliquidon	stimulates insulin secretion from the β-cells of the islets of Langerhans increases insulin and C-peptide secretion	second generation sulphonylureas are superior to first generation finding not proven in CVOTs	gliclazide only: ↑EPC (CD34+/VEGFR2+/CD45-/dim) count assessed by flow cytometry ↑FMD	[118]
			no proven effects for other sulphonylureas	[119,120]
Thiazolidinediones: Pioglitazone Rosiglitazone	reduces insulin resistance and reduces insulin concentrations activates peroxisome proliferator-activated receptor gamma increases insulin sensitivity of liver, fat, and skeletal muscle cells reduces hepatic glucose output increases peripheral glucose disposal	improves some MACEs not proven in CVOTs increased risk of heart failure	pioglitazone: ↑EPC (CD34+/VEGFR2+) count assessed by flow cytometry pioglitazone:	[121]
			↑ circulating CD34+ cell count	[122]
			pioglitazone: ↑EPC (CD34+) count assessed by flow cytometry ↑increased migratory response and adhesion capacity to fibronectin and collagen in culture	[123]
			pioglitazone: ↑EPC (CD34+/VEGFR2+) count assessed by flow cytometry ↑ SDF1 induced migratory capacity ↑ ECFC in cultures	[124]
			pioglitazone: no effect on EPC count	[125]
DPP-4 inhibitors Sitagliptin Linagliptin Alogliptin Saxagliptin Vildagliptin Teneligliptin	inhibits dipeptidyl peptidase 4 (DPP-4) enhances the levels of glucagon-like peptide-1 (GLP-1) and glucose-dependent insulinotropic polypeptide (GIP) in a glucose-dependent manner improves beta cell responsiveness to glucose and stimulates insulin biosynthesis and release lowers glucagon secretion reduces hepatic glucose production	non-inferiority to standard treatment/class effect proven in CVOTs except for vildagliptin and teneligliptin increased risk for heart failure for saxagliptin	sitagliptin: ↑EPC (CD34+/VEGFR2+) count assessed by flow cytometry ↑ SDF1 blood concentrations	[126]
			sitagliptin: ↑EPC (CD34+/CXCR4+) count assessed by flow cytometry ↓ SDF1 blood concentrations	[120]
			sitagliptin: ↑EPC (CD34+/VEGFR2+ and CD34+/VEGFR2+/CD133+) count assessed by flow cytometry ↑GLP-1, NO and SDF-1 blood concentrations	[127]
			sitagliptin: ↑EPC (CD34+) count assessed by flow cytometry ↑FMD	[128]
			linagliptin: ↑EPC (CD34+/VEGFR2+ and CD34+/CD133+) count ↑GLP-1, and SDF-1 blood concentrations	[129]
			linalgiptin no effect on EPCs ↑SDF-1 blood concentrations	[130]
			linagliptin↑CD34+/CD184+ EPC improved arterial stiffness and pulse wave velocity	[131]
			vildagliptin: ↑EPC (CD34+/VEGFR2 +/CD133+) count assessed by flow cytometry ↓SDF1 blood concentrations	[119]
			alogliptin: ↑EPC (CD34+/VEGFR2 +/CD45-/dim) count assessed by flow cytometry	[132]
			saxagliptin: ↑EPC (CD34+/VEGFR +/CD133+) count assessed by flow cytometry ↑FMD	[133]
			saxagliptin: no effect on EPC if added to metformin improved migratory capacity	[134]
			teneligliptin: no significant effect on EPC ↑FMD	[135]
GLP-1 receptor agonists Exenatide Lixisenatide Liraglutide Dulaglutide Semaglutide	activates the GLP-1 receptor stimulates insulin secretion and lowers glucagon secretion in a glucose-dependent manner delays gastric emptying in the early postprandial phase.	liraglutide, dulaglutide, and semaglutide showed cardiovascular superiority in CVOTs or equivalent studies	exenatide in full dose superior to medium dosed liraglutide in ↑EPC (CD34+/VEGFR2 +)	[136]
			liraglutide superior to sitagliptin in ↑VEGF and SDF-1	[137]
			dulaglutide: ↑EPC (CD34+/VEGFR2 +/CD133+) enhanced EPC proliferation, adhesion, migration, and tubule formation abilities improved brachial-ankle pulse wave velocity	[138]
SGLT-2 inhibitors Empagliflozin Dapagliflozin Canagliflozin	inhibits the sodium-glucose cotransporter-2 by dapagliflozin in the proximal renal tubule improves both fasting and postprandial plasma glucose levels	cardiovascular superiority proven in CVOTs beneficial in heart failure and renal failure black box warning for lower limb amputations	dapagliflozin lead to a late increase in EPC count (CD34+/VEGFR2+) no change in EPCs in the empagliflozin group	[139]
			dapagliflozin treatment activated AMPK signaling in EPCs	[140]
			empagliflozin increases CD133+EPC count	[141]
			canagliflozin improved CXCR receptors on EPCs, improving their migratory capacity	[142]
Insulin and insulin analogs	binds to insulin receptors	proven non-inferiority to standard therapy in CVOTs for newer insulin analogs	detemir and glargin both increased EPC count (CD34+/VEGFR2+ and CD34+/VEGFR2+/CD133+) decreased adhesion molecules	[143]
			no difference in CD34*/VEGFR2+ EPCs between groups receiving NPH insulin, insulin glargin, or oral therapy improved ECFC growth with NPH insulin and glargin decrease in intima media thickness	[144]
			intensive insulin therapy increased EPC count (CD34+/VEGFR2+) no effect on clinical outcomes	[145]
			reduced glucovariability by using insulin pumps increased the EPC count (CD34+/VEGFR+) compared to intensified insulin therapy	[58] *

* indicates studies of patients with type 1 diabetes, ↑ increased, ↓ reduced.
